# Coexpression of PD-1, 2B4, CD160 and KLRG1 on Exhausted HCV-Specific CD8+ T Cells Is Linked to Antigen Recognition and T Cell Differentiation

**DOI:** 10.1371/journal.ppat.1000947

**Published:** 2010-06-10

**Authors:** Bertram Bengsch, Bianca Seigel, Marianne Ruhl, Jörg Timm, Martin Kuntz, Hubert E. Blum, Hanspeter Pircher, Robert Thimme

**Affiliations:** 1 Department of Medicine II, University of Freiburg, Freiburg, Germany; 2 Spemann Graduate School of Biology and Medicine (SGBM), University of Freiburg, Freiburg, Germany; 3 Faculty of Biology, University of Freiburg, Freiburg, Germany; 4 Department of Virology, University of Essen, Essen, Germany; 5 Department of Immunology, University of Freiburg, Freiburg, Germany; Nationwide Children's Hospital, United States of America

## Abstract

Exhausted CD8+ T cell responses during chronic viral infections are defined by a complex expression pattern of inhibitory receptors. However, very little information is currently available about the coexpression patterns of these receptors on human virus-specific CD8+ T cells and their correlation with antiviral functions, T cell differentiation and antigen recognition. We addressed these important aspects in a cohort of 38 chronically HCV infected patients and found a coexpression of inhibitory receptors such as 2B4, CD160 and KLRG1 in association with PD-1 in about half of the HCV-specific CD8+ T cell responses. Importantly, this exhaustive phenotype was associated with low and intermediate levels of CD127 expression, an impaired proliferative capacity, an intermediate T cell differentiation stage and absence of sequence variations within the corresponding epitopes, indicating ongoing antigen triggering. In contrast, a low expression of inhibitory receptors by the remaining HCV-specific CD8+ T cells occurred in concert with a CD127hi phenotype, an early T cell differentiation stage and presence of viral sequence variations within the corresponding epitopes. In sum, these results suggest that T cell exhaustion contributes to the failure of about half of HCV-specific CD8+ T cell responses and that it is determined by a complex interplay of immunological (e.g. T cell differentiation) and virological (e.g. ongoing antigen triggering) factors.

## Author Summary

About 170 million people are infected with hepatitis C virus (HCV), which may cause severe liver disease and liver cancer. Upon acute infection, only about 30% of patients are able to eliminate the virus spontaneously while about 70% of patients develop chronic infection. It is known that a successful immune response against HCV depends on virus-specific CD8+ T cells. However, during chronic infection, these cells are impaired in their antiviral function. In this study, we found that the exhaustion is characterized by the expression of multiple inhibitory receptors, such as PD-1, 2B4, CD160 and KLRG1. Of note, the coexpression of these receptors depends on the ongoing recognition of the viral antigen and the maturation stage of the T cell. The remaining virus-specific T cell responses that are not exhausted do not recognize the virus present in the patients any more due to viral mutations, indicating viral escape. Thus, they fail to exert antiviral activity, although they share characteristics of fully functional memory T cells. In sum, we have found that T cell exhaustion contributes to the failure of about half of HCV-specific CD8+ T cell responses and that it is determined by a complex interplay of immunological and virological factors. These findings will be important to consider in the design of new antiviral vaccination strategies.

## Introduction

Virus-specific CD8+ T cells play a central role in the outcome of HCV infection. Indeed, several human and animal studies have shown associations between strong and multispecific T cell responses and viral clearance [1]. During chronic HCV infection, viral escape and an impairment of HCV-specific CD8+ T cell antiviral functions, e.g. the ability to proliferate or to secrete antiviral cytokines such as interferon-γ (IFN-γ) contribute to virus-specific CD8+ T cell failure. The underlying mechanisms for the functional impairment of HCV-specific CD8+ T cells have not been clarified in detail, although lack of CD4+ T cell help, action of regulatory T cells and expression of immunomodulatory cytokines, such as Il-10, have been suggested to contribute [1]. In addition, expression of the inhibitory receptor PD-1 has been postulated to characterize a state of exhaustion of HCV-specific CD8+ T cells in chronic HCV infection in analogy to murine models of chronic viral infections [2]. Indeed, analysis of patients with chronic HCV infection identified high levels of PD-1 expression on HCV-specific CD8+ T cells in blood and liver [3] and blockade of PD-1 signaling resulted in the functional restoration of blood-derived HCV-specific CD8+ T cell responses in chronic infection [3], [4]. However, the relevance of PD-1 in defining exhausted HCV-specific CD8+ T cells has not been unchallenged. For example, PD-1 blockade alone was unable to restore the function of liver-derived HCV-specific CD8+ T cells [5] while targeting additional inhibitory signaling pathways reinvigorated the antiviral function [6]. In addition, PD-1 expression did not necessarily identify exhausted HCV-specific CD8+ T cells during acute HCV infection in humans [7] and chimpanzees [8]. Thus, PD-1 expression alone may not be sufficient to determine exhaustion of HCV-specific CD8+ T cells during HCV infection. In this context, it is interesting to note that a recent study identified coexpression of additional inhibitory receptors next to PD-1 as a critical determinant of CD8+ T cell exhaustion in a murine model of chronic viral infection. For example, expression of several inhibitory receptors including 2B4 and CD160 next to PD-1 was identified on strongly exhausted virus-specific CD8+ T cells in severe LCMV infection [9]. 2B4 is a coregulatory receptor of the SLAM-receptor family that is capable of mediating both activatory and inhibitory signals upon ligand CD48 binding [10]. High levels of 2B4 expression are associated with 2B4 inhibitory receptor function [10] and indeed, 2B4 and CD160, a glycoylphosphatidylinositol-anchored receptor that coinhibits T cells upon ligand HVEM binding [11], both highly correlated in gene expression analysis with PD-1 expression [12]. The positive correlation with PD-1 expression was also observed for other inhibitory receptors, such as LAG-3, a protein closely related to CD4, and CTLA-4, a member of the CD28 family of receptors [9]. Of note, expression of the inhibitory receptor Killer-cell-lectin-like receptor G1 (KLRG1) showed no correlation with exhaustion in severe LCMV infection [9]. KLRG1 mediates proliferative dysfunction of differentiated CD8+ T cells upon ligand E-cadherin binding [13], [14], [15] and its upregulation is thought to be mediated by ongoing antigen triggering [16].

Despite these insights into inhibitory receptor expression on CD8+ T cells, very little information is currently available about the coexpression of these receptors on virus-specific CD8+ T cells during chronic human infection such as HCV and their association with T cell function, differentiation and antigen recognition. Here, we show that the coexpression of inhibitory receptors such as PD1, 2B4, CD160 and KLRG1 occurs in about half of HCV-specific CD8+ T cell responses and that it is linked to low or intermediate levels of CD127 expression, impaired proliferative capacity, an intermediate T cell differentiation stage and ongoing antigen-recognition. In contrast, the absence of inhibitory receptor expression is associated with a CD127 high phenotype and the presence of virus sequence variations in the respective epitopes, indicating viral escape. These results indicate that two different mechanisms contribute to the dysfunction of the HCV-specific CD8+ T cell pool in chronic HCV infection. These results also indicate that different factors are involved in the development of HCV-specific CD8+ T cell exhaustion.

## Results

### Expression of inhibitory receptors on HCV-specific CD8+ T cells

**Figure 1:**
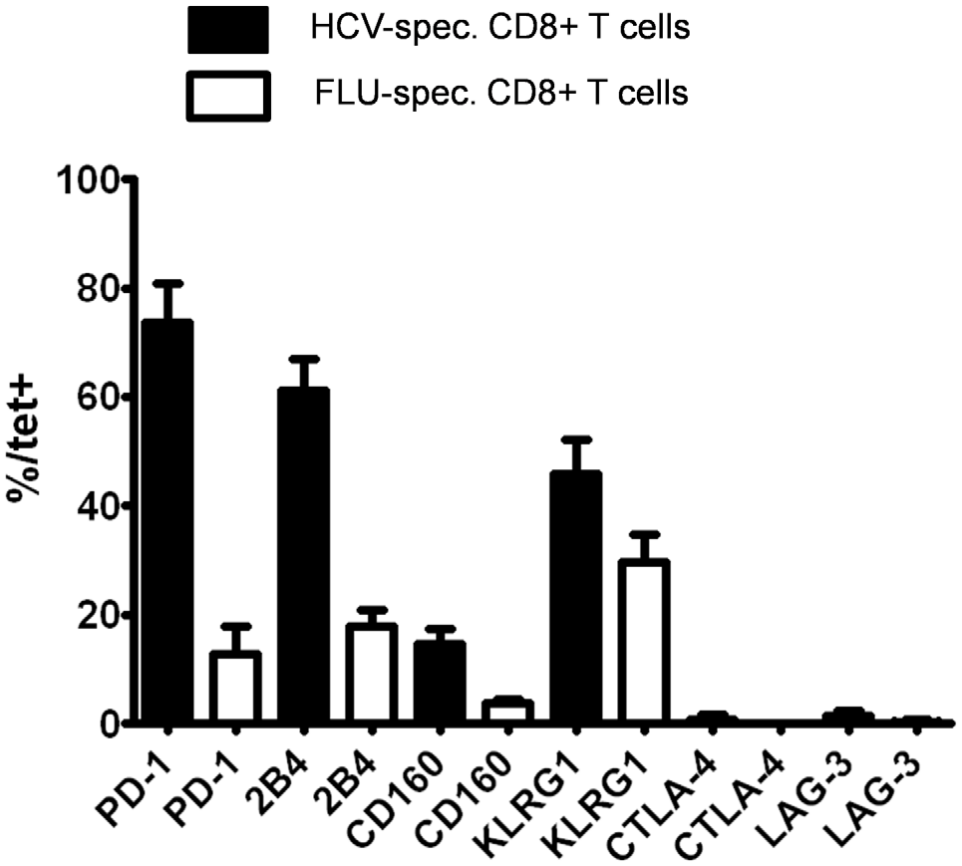
Expression of various inhibitory receptors by HCV-specific CD8+ T cells. The expression of PD-1, 2B4, CD160, KLRG1, CTLA-4 and LAG-3 was monitored on HCV-specific CD8+ T cell responses targeting multiple immunodominant HCV epitopes of 10 consecutive patients and compared to the expression of these receptors on virus-specific CD8+ T cells targeting an immunodominant Influenza matrix epitope. Mean expression and SEM of each inhibitory receptor on virus-specific CD8+ T cells is shown.

**Figure 2:**
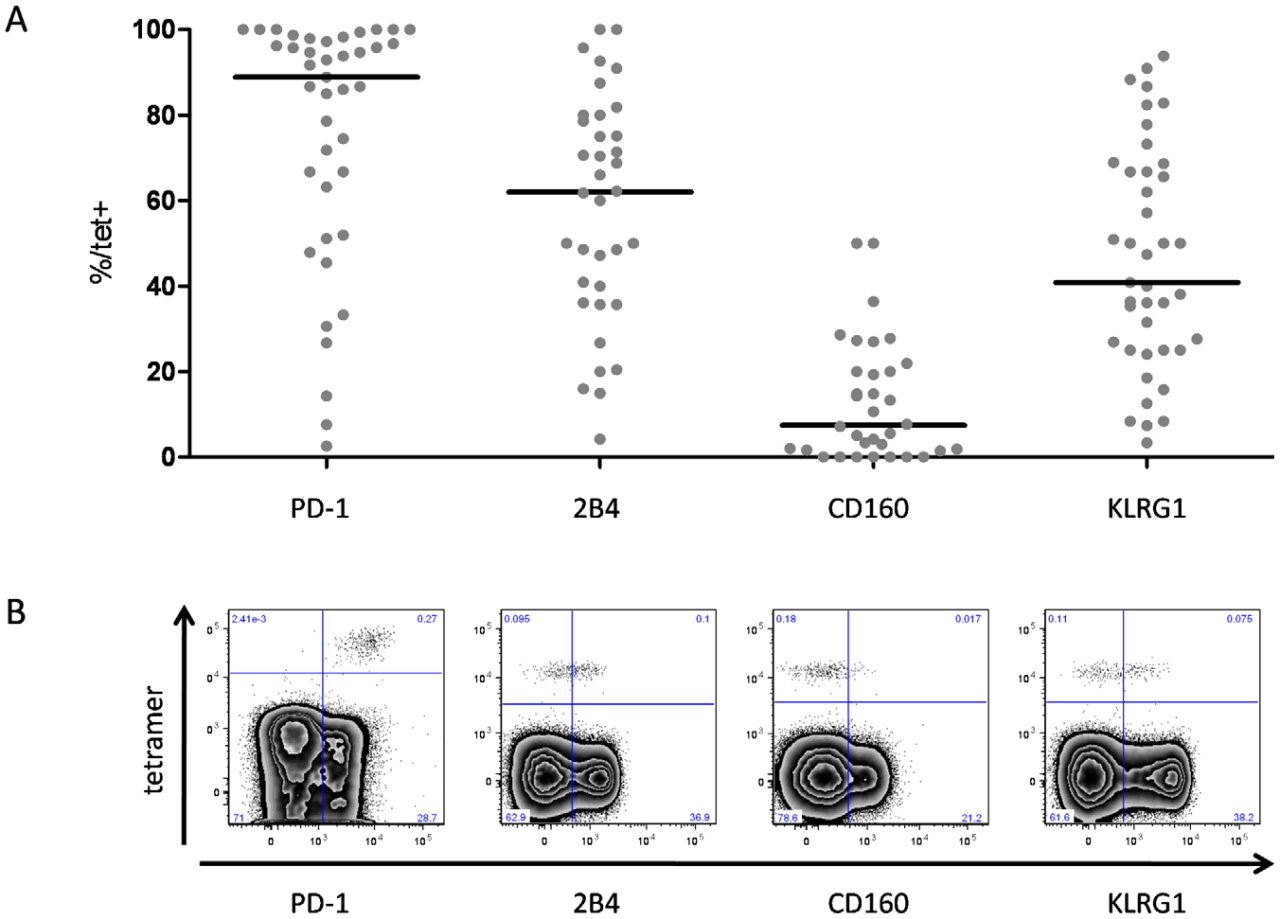
Expression of PD-1, 2B4, CD160 and KLRG1 on HCV-specific CD8+ T cells. (A) Frequency of inhibitory receptor expression on HCV-specific CD8+ T cells. Each dot represents inhibitory receptor expression on epitope-specific CD8+ T cells. Median expression of inhibitory receptors in the study cohort was 88,9% (PD-1), 62,0% (2B4), 7,4% (CD160), and 40,8% (KLRG1). (B) Representative tetramer stainings chosen to depict approximately the median inhibitory receptor expression of the total cohort. Plots are displayed using biexponential transformation (FlowJo 8.8.6).

In a first set of experiments, we screened HCV-specific CD8+ T cells for the expression of a large set of inhibitory receptors such as PD-1, 2B4, CD160, KLRG1, LAG-3 and CTLA-4 in a cohort of 10 chronically HCV infected patients. We compared the expression of these receptors on HCV-specific CD8+ T cells with the expression on FLU-specific CD8+ T cells that represent a memory population. As shown in Fig. 1, we found an elevated expression of PD-1, 2B4, CD160 and KLRG1 by HCV-specific CD8+ T cells, however, no significant increase of LAG-3 and CTLA-4 expression was observed. Based on these results, we focused in the following on the analysis of the expression of PD-1, 2B4, CD160 and KLRG1 by HCV-specific CD8+ T cells in a cohort of 38 patients with chronic HCV that displayed HCV-specific tetramer responses (Table 1). As shown in Fig. 2A, we found a high expression of PD-1 in our cohort (median: 88.9%). Although PD-1 expression was high on most HCV-specific CD8+ T cell responses, it was not detectable on all HCV-specific CD8+ T cells (Fig. 2). 2B4 was expressed by some HCV-specific CD8+ T cells with a median expression of 62.0% (Fig. 2A and B), whereas CD160 was detectable only on a fraction of virus-specific CD8+ T cells (median: 7.4%) (Fig. 2A and B). Expression of KLRG1 was found to be quite variable, with a median expression of 40.8% (Fig. 2A and B).

**Table 1:** Characteristics of the study population.

Patient	Sex	Age	gt	viral load (IU/ml)	ALT (U/l)	Liver histology
1	m	67	1b	n.a.	90	°2/°2
2	f	70	1b	*7208937*	60	*°2/°3–4*
3	m	52	1b	1409300	85	n.a.
4	m	30	1a	81230	39	°1/°0
5	m	46	2b	400288	249	°2/°3
6	m	25	1a	729658	80	°1/°1
7	m	42	1a	4679963	235	°2/°3
8	f	65	5	1558856	65	°2/°2
9	f	54	1b	*208729*	45	°1/°2
10	m	53	3a	*2096500*	147	°2/°2
11	m	38	3a	438607	41	°2/°1
12	m	32	*3a*	*7691548*	*515*	°2–3/°2–3
13	f	40	1b	1400000	35	°1/°0
14	f	69	1b	*297000*	60	°1/°2
15	m	51	1a	1201680	98	°2/°2–3
16	f	44	1a	685910	62	n.a.
17	f	70	1a	*1887862*	219	°2/°2
18	f	63	1b	2568188	104	°1/°2
19	f	55	3a	819293	68	°3/°3
20	m	31	1a	791341	287	°1/°1
21	f	57	1b	3160130	101	°1/°3
22	f	25	1b	50658	112	°1/°0
23	f	67	1b	427220	34	°1/°2
24	f	30	1b	37499	54	°0/°0
25	m	58	1a	3802978	177	*°1/°3*
26	f	48	1a	363636	43	n.a.
27	m	28	1b	78947	33	°2/°1
28	m	40	1a	382481	163	°2/°1
29	m	54	1a	3185390	133	°2/°3
30	f	61	1b	1537679	184	°2/°1
31	f	55	1b	995000	40	°3/°1
32	f	43	1b	171803	34	*°0/°0*
33	f	56	1a	938114	91	°2/°1
34	m	36	1a	4999910	75	°1/°2
35	m	40	1a	4011231	43	°2/°2
36	m	41	1a	1360020	65	°3/°2
37	f	54	1b	297322	65	°1/°1
38	f	45	1a	1316760	25	*°0/°1*

Liver biopsies were rated for degree of inflammation/degree of fibrosis according to the METAVIR score. Abb. f, female; gt, HCV genotype; m, male; n.a., not available.

### Expression of inhibitory receptors is associated with low levels of CD127 expression on HCV specific CD8+ T cells

**Figure 3:**
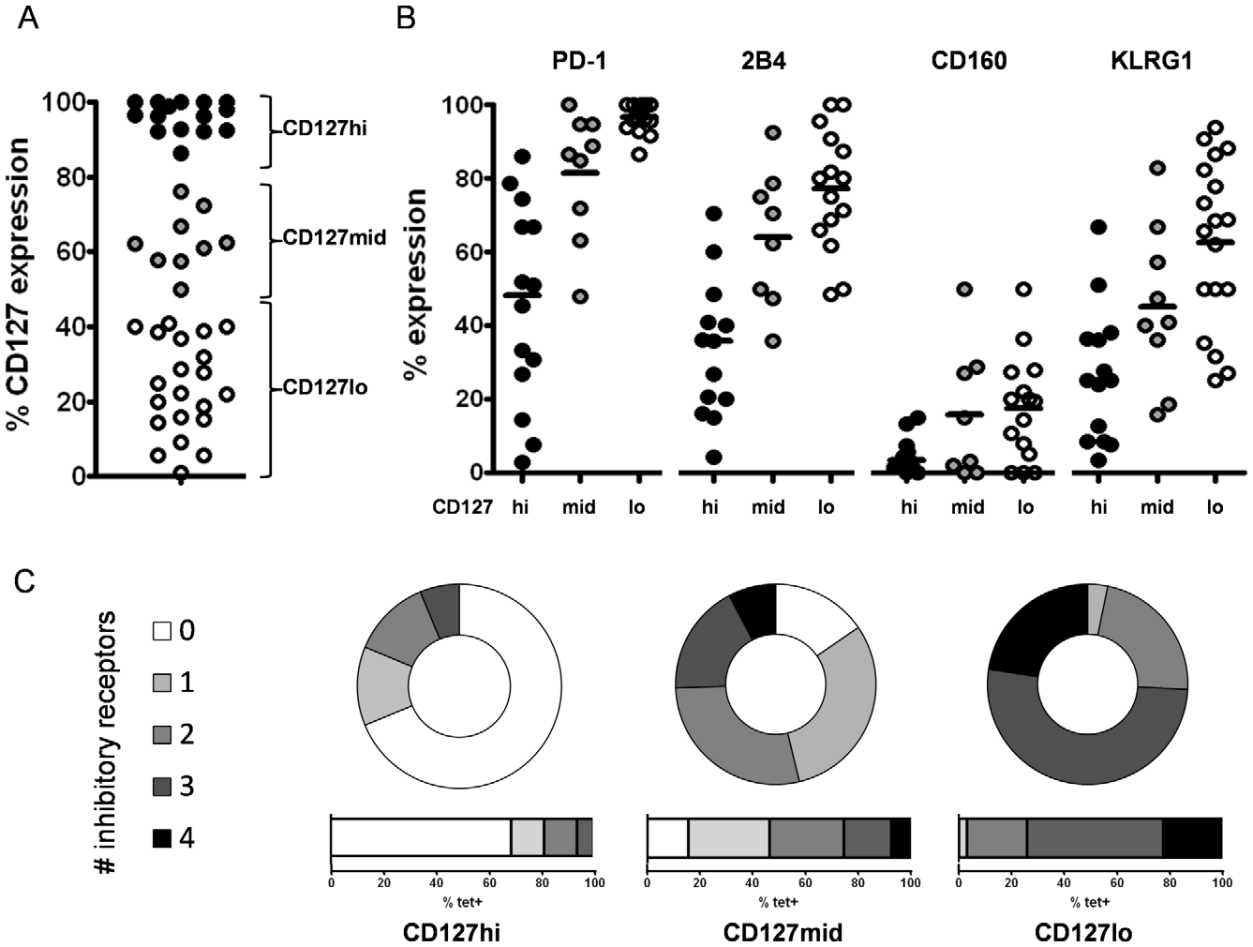
Inhibitory receptor expression is inversely linked to level of CD127 expression. (A) Three groups of HCV-specific CD8+ T cell responses can be distinguished by level of CD127 expression. High levels of CD127 expression (>80%) are displayed by black circles, intermediate levels of CD127 (50–80%) by grey circles and a low expression of CD127 (<50%) by white circles. (B) Expression of inhibitory receptors PD-1, 2B4, CD160 and KLRG1 is shown for CD127hi (black circles), CD127mid (grey circles) and CD127lo (white circles) HCV-specific CD8+ T cells. Significant differences of inhibitory receptor expression between the CD127 subgroups were observed for PD-1 expression (p<0.0001), 2B4 expression (p<0.0001), CD160 expression (p<0.05) and KLRG1 expression (p<0.0001). (C) Coexpression of inhibitory receptors on HCV-specific CD8+ T cells on a single-cell-level. Displayed is the frequency of HCV-specific CD8+ T cells within a given epitope expressing 0 (white), 1 (light grey), 2 (grey), 3 (dark grey) or 4 (black) inhibitory receptors. Representative diagrams are shown for a CD127hi epitope (pt. 4-NS3-1406), CD127mid epitope (pt. 26-NS3-1406) and CD127lo epitope (pt. 4-NS5-2594). Coexpression of inhibitory receptors inversely correlates with CD127 expression.

Previously, we have shown that HCV-specific CD8+ T cells consist of subsets with distinct phenotypical and functional properties that can be defined by CD127 expression [17]. Thus, we asked whether the expression of CD127 is also linked to the expression of the inhibitory receptors PD-1, 2B4, CD160 and KLRG1 by HCV-specific CD8+ T cells. To address this question, we costained the inhibitory receptors and CD127 on HCV-specific CD8+ T cells. Depending on the expression level of CD127 (Fig. S1), we defined three groups of HCV-specific CD8+ T cells as displayed in Fig. 3A: a CD127 high (hi) group with more than 80% of epitope-specific CD8+ T cells expressing CD127, a CD127 intermediate (mid) group with 50–80% of CD127 expression and a CD127 low (lo) group with less than 50% expression of CD127. Importantly, we found a clear association between the expression of CD127 and the expression of inhibitory receptors on HCV-specific CD8+ T cells. Indeed, CD127lo cells highly expressed PD-1 (median 97.5%), 2B4 (median 80.0%) and CD160 (median 19.3%) (Fig. 3B). CD127mid cells expressed slightly reduced levels of PD-1 (median 86.7%), 2B4 (median 66.4%) and CD160 (median 8.9%). In contrast, CD127hi cells showed a significantly lower expression of PD-1 (median 48.3%), 2B4 (median 35.7%) and CD160 (median 3.3%) (Fig. 3B). KLRG1 was also higher expressed on HCV-specific CD8+ T cells with a CD127lo phenotype compared to a CD127mid and CD127hi phenotype (67.1% vs 40.8% vs 25.0%) (Fig. 3B). In contrast to PD-1, 2B4 and CD160, however, the range of KLRG1 expression varied more strongly between both groups. Next, we analyzed the coexpression of inhibitory receptors on HCV-specific CD8+ T cells in 10 patients at the single cell level in a multi-inhibitory receptor staining (Fig. S2). As shown in Fig. 3C, a CD127lo phenotype was associated with a high frequency of HCV-specific CD8+ T cells coexpressing multiple inhibitory receptors. The inhibitory receptor coexpression profile of CD127mid HCV-specific CD8+ T cells was reduced compared to CD127hi cells. For example, in our cohort, more than 80% of CD127lo cells coexpressed 2 or more inhibitory receptors while coexpression was found in less than 55% of CD127mid cells (Fig. 3C). In contrast, a CD127hi phenotype was associated with low or absent levels of inhibitory receptor coexpression. These results clearly illustrate that the coexpression of multiple inhibitory receptors by HCV-specific CD8+ T cells is linked to low levels of CD127 expression.

### Coexpression of inhibitory receptors on CD127lo HCV-specific CD8+ T cells correlates with impaired proliferative capacity

**Figure 4:**
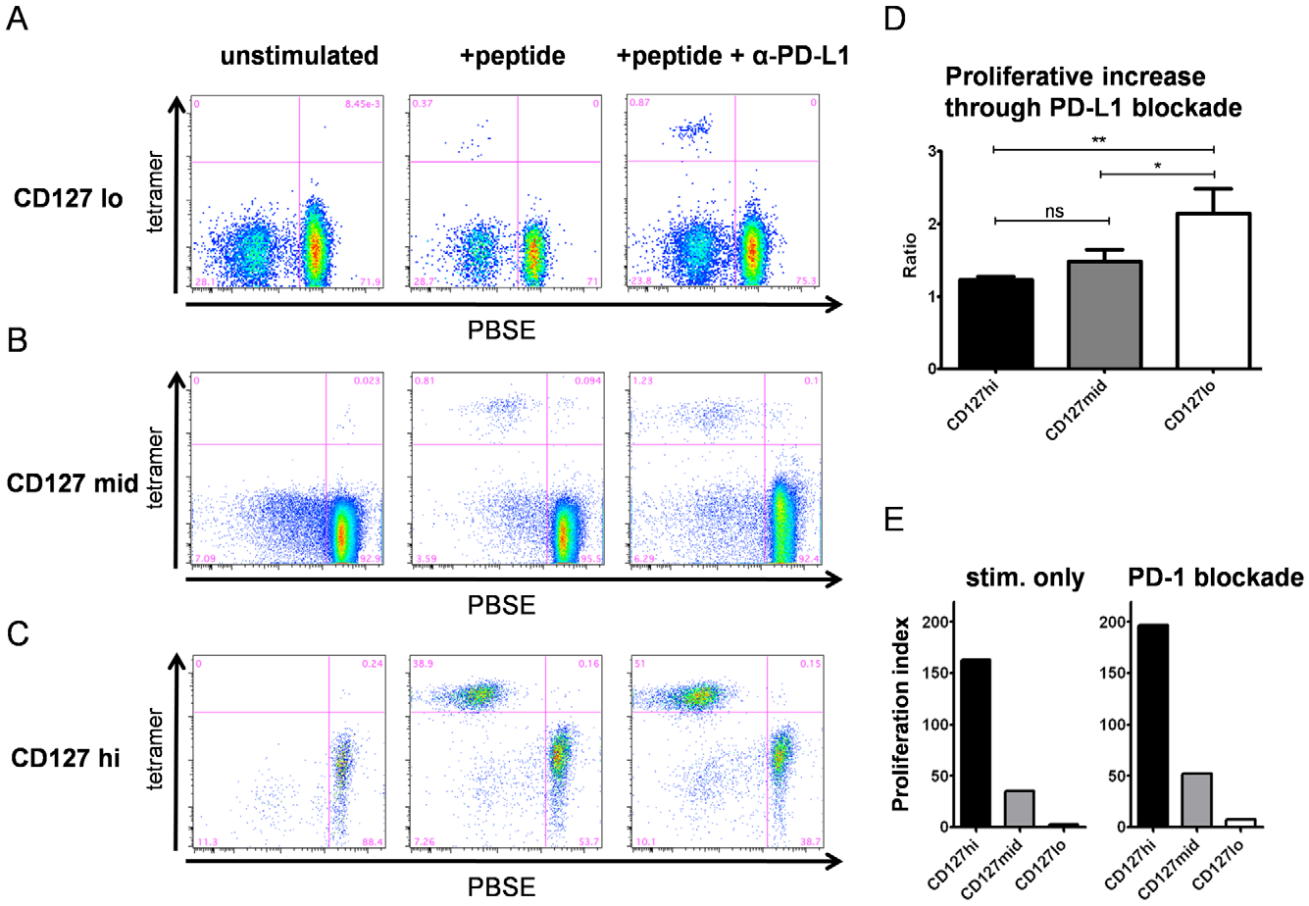
Proliferative capacity and exhaustion inversely correlate with CD127 expression. Proliferation of HCV-specific CD8+ T cells upon peptide stimulation in the presence or absence of PD-L1 blockade was measured by reduction in PBSE fluorescence in short term cultures of 15 patients. Representative stainings are shown for (A) CD127lo (pt.4-NS5-2594), (B) CD127mid (pt. 13-NS3-1073) and (C) CD127hi (pt. 32-NS5-2594) HCV-specific CD8+ T cells. (D) Comparison of the increase in proliferation depending on PD-L1 blockade. Depicted are the mean values and SEM for the ratio (frequency of HCV-specific CD8+ T cells after peptide stimulation and PD-L1 blockade/frequency of HCV-specific CD8+ T cells after peptide stimulation). This ratio reflects the effect of PD-L1 blockade in addition to peptide stimulation. PD-L1 blockade in the cohort analyzed was significantly more effective for peptide-stimulated CD127lo cells (mean 2.2) than for CD127mid (mean 1.5) and CD127hi cells (mean 1.2) (p<0.01). (E) Proliferation index of peptide-stimulated HCV-specific CD8+ T cells in the presence (right side) and absence (left side) of PD-L1 blockade. The proliferation index (PI) was calculated by the formula (frequency of HCV-specific CD8+ T cells after culture/frequency of HCV-specific CD8+ T cells ex vivo) and reflects the proliferative capacity of the cell, which was highest for CD127hi HCV-specific CD8+ T cells compared to CD127mid and CD127lo cells.

Next, we asked whether the coexpression of multiple inhibitory receptors on CD127lo CD8+ T cells defines functionally impaired HCV-specific CD8+ T cells, indicating exhaustion. We addressed this by stimulating CD127lo, CD127mid and CD127hi HCV-specific CD8+ T cells antigen-specifically for one week and by analyzing the subsequent proliferation in the presence or absence of PD-L1 blockade. Of note, we found significant differences regarding the proliferative capacity and the effect of PD-L1 blockade between these groups (Fig. 4). Indeed, the proliferative capacity of CD127hi HCV-specific CD8+ T cells was much higher upon antigen stimulation compared to CD127mid and CD127lo HCV-specific CD8+ T cells. As shown in Fig. 4A and 4E, CD127lo HCV-specific CD8+ T cells that expressed multiple inhibitory receptors proliferated poorly upon antigen stimulation. However, these cells could be reinvigorated partially upon blockade of the inhibitory PD-1/PD-L1 signaling pathway, indicating that they were in a functional reversible state of exhaustion. CD127mid HCV-specific CD8+ T cells displayed an improved proliferative capacity upon antigen stimulation (Fig. 4B). In contrast to CD127lo and CD127mid cells, CD127hi HCV-specific CD8+ T cells proliferated vigorously (Fig. 4C) upon stimulation with peptide alone. Additional PD-L1 blockade resulted in only a minor increase in proliferation. In a subsequent analysis of 15 patients, we found that the increase in HCV-specific CD8+ T cells due to PD-L1 blockade was about 2.2fold for CD127lo cells. This increase was significantly higher compared to CD127mid (1.5fold) and CD127hi cells (1.2fold) (p<0.01) (Fig. 4D). These results indicate that CD127lo HCV-specific CD8+ T cells are functionally impaired *in vivo* and are functionally dependent on inhibitory receptor blockade, indicating exhaustion.

### The expression of specific inhibitory receptors is linked to stage of T cell differentiation

**Figure 5:**
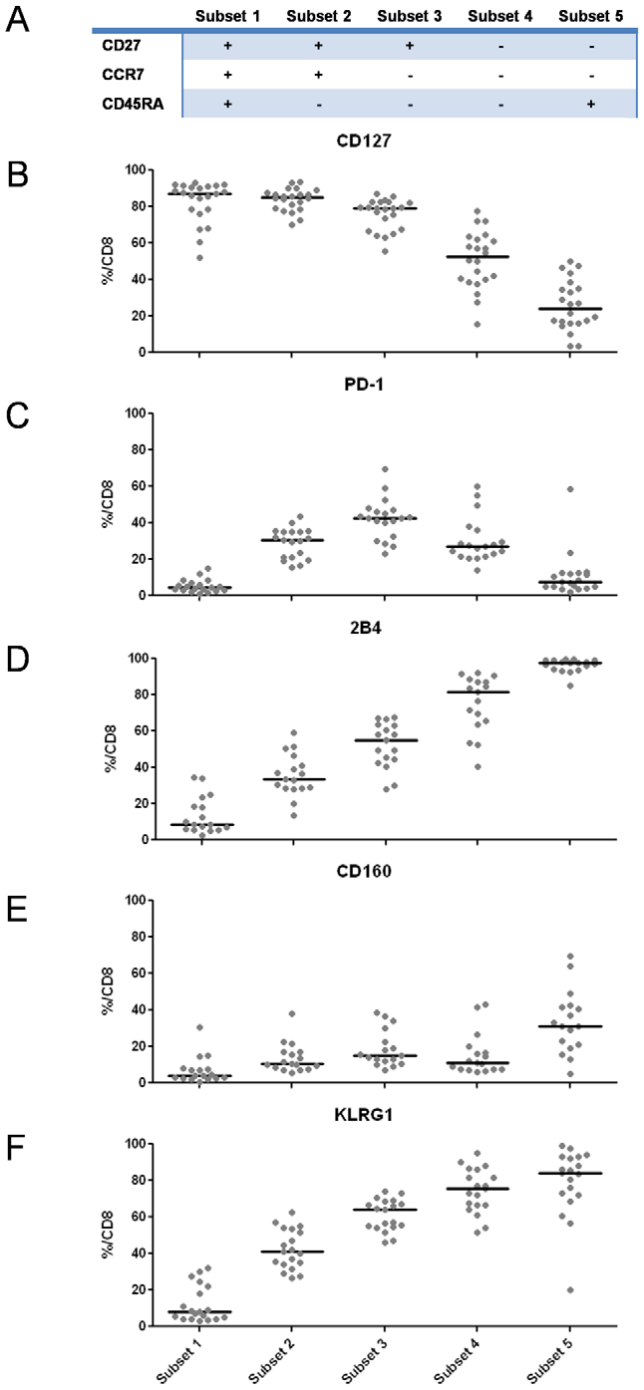
Receptor expression profiles are linked to T cell differentiation. (A) Schematic overview of the differentiation stage of CD8+ T cells assessed by differential CD27, CCR7 and CD45RA expression. (B–F) Expression of CD127 and inhibitory receptors PD-1, 2B4, CD160 and KLRG1 in individual CD8+ T cell differentiation subsets. Each dot represents the frequency of CD127 or inhibitory receptor expression on CD8+ T cells belonging to the given differentiation subset of an individual patient.

**Figure 6:**
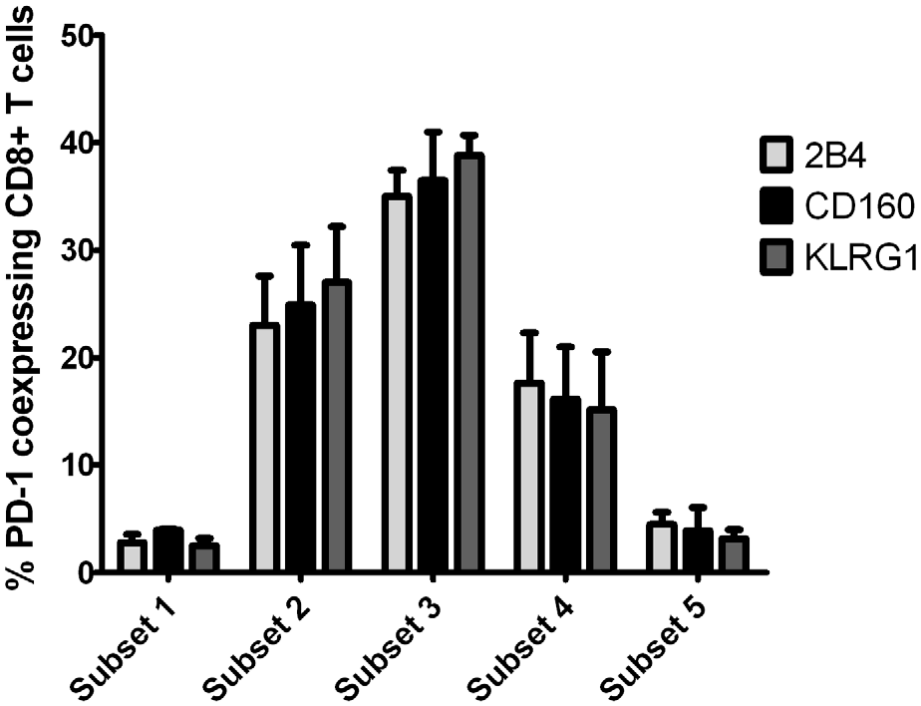
Coexpression of 2B4, CD160 and KLRG1 with PD-1 is linked to an intermediate differentiation stage. Frequency of CD8+ T cells coexpressing inhibitory receptors 2B4, CD160 and KLRG1 next to PD-1 is shown depending on the differentiation stage as defined in Fig. 5A. Values were calculated by dividing the number of coexpressing CD8+ T cells belonging to the 5 differentiation subsets analyzed by the total number of coexpressing CD8+ T cells. A total of 5 patients were analyzed.

It has previously been shown that the expression of inhibitory CD8+ T cell receptors, e.g. PD1 is linked to the stage of T cell differentiation [18], [19]. Thus, we asked whether the coexpression of CD127 and all inhibitory receptors analyzed in our study was linked to certain stages of T cell differentiation. For this analysis, we assessed post-thymic human CD8+ T cell differentiation using a linear model based on CD27, CCR7 and CD45RA expression as displayed in Fig. 5A [18]. CD127 expression was highest in naïve and early T cell subsets (subsets 1 and 2), but reduced in late differentiated CD8+ T cells, indicating that CD127 expression was linked to early stages of T cell differentiation (Fig. 5B). In agreement with a previous study, the expression of PD-1 was highest on intermediate differentiated CD8+ T cells (subset 3) and lowest on naïve and late differentiated CD8+ T cells [19] (Fig. 5C). Interestingly, the expression patterns of 2B4, CD160 and KLRG1 did not mirror the expression pattern of PD-1 but displayed unique, individual profiles. 2B4 expression was lowest on naïve CD8+ T cells but progressively increased along the differentiation subsets reaching almost 100% expression on late-differentiated CD8+ T cells (Fig. 5D). CD160 expression was lowest in naïve T cells and increased in further differentiated subsets with two expression peaks in intermediate subset 3 and late differentiated T cell subset 5 (Fig. 5E) while KLRG1 showed a similar expression pattern as 2B4 being low in naïve and early subsets but enriched in late differentiated subsets (Fig. 5F). Overall, these results indicate that inhibitory receptors are increasingly expressed by further differentiated CD8+ T cells. However, since very distinct patterns of expression were observed for each marker, the expression of one inhibitory receptor does not necessarily indicate coexpression of others. Thus, we asked whether coexpression of the inhibitory receptors 2B4, CD160 and KLRG1 together with PD-1 occurred in a specific T cell differentiation subset. Indeed, as shown in Fig. 6, we identified the highest level of coexpression of all markers in the intermediate T cell subset 3. These results indicate that inhibitory receptors exhibit individual and distinct expression patterns linked to T cell differentiation and that coexpression of 2B4, CD160 and KLRG1 with PD-1 indicating exhaustion occurs in intermediate rather than late differentiated CD8+ T cell subsets.

**Figure 7:**
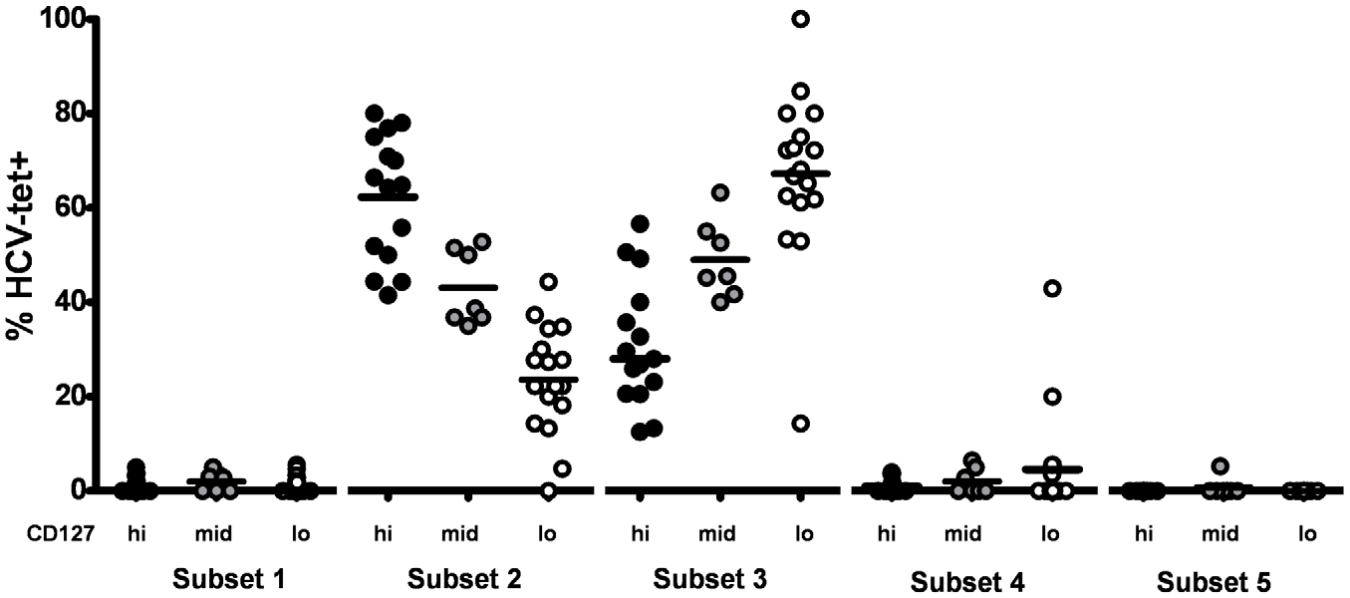
Differences in HCV-specific CD8+ T cell differentiation depending on CD127 expression. The frequency of HCV-specific CD8+ T cells identified in the differentiation subsets defined in Fig. 5A is depicted for CD127hi (Black circles), CD127mid (grey circles) and CD127lo (white circles) responses. Significant differences between CD127 subgroups were observed for differentiation subset 2 (p<0.0001) and subset 3 (p<0.0001). The majority of CD127hi responses was found in subset 2 (median 64,8%) while the majority of CD127lo cells was observed in subset 3 (median 68,1%). CD127mid cells were distributed at comparable levels in subset 2 (median 38,7%) and subset 3 (median 45,7%).

Finally, we asked whether the same association can also be found within the HCV-specific CD8+ T cell population. As shown in Fig. 7 and in agreement with previous findings, we observed that HCV-specific CD8+ T cells consisted mostly of early and intermediate differentiated T cells. Interestingly, the differentiation of HCV-specific CD127hi, CD127mid and CD127lo cells was significantly different (p<0.0001). The majority of CD127hi cells belonged to the early differentiation subset 2, whereas CD127mid cells were distributed at comparable levels in subset 2 and subset 3. In contrast, HCV-specific CD127lo cells were prominently found within the intermediate subset 3. Keeping in mind that the coexpression of inhibitory receptors is highest in the intermediate subset 3 and associated with a CD127lo phenotype, these results clearly suggest that there is a complex link between CD127 expression, T cell differentiation and T cell exhaustion.

### Expression of inhibitory receptors on CD127lo CD8+ T cells is dependent on ongoing antigen recognition

**Figure 8:**
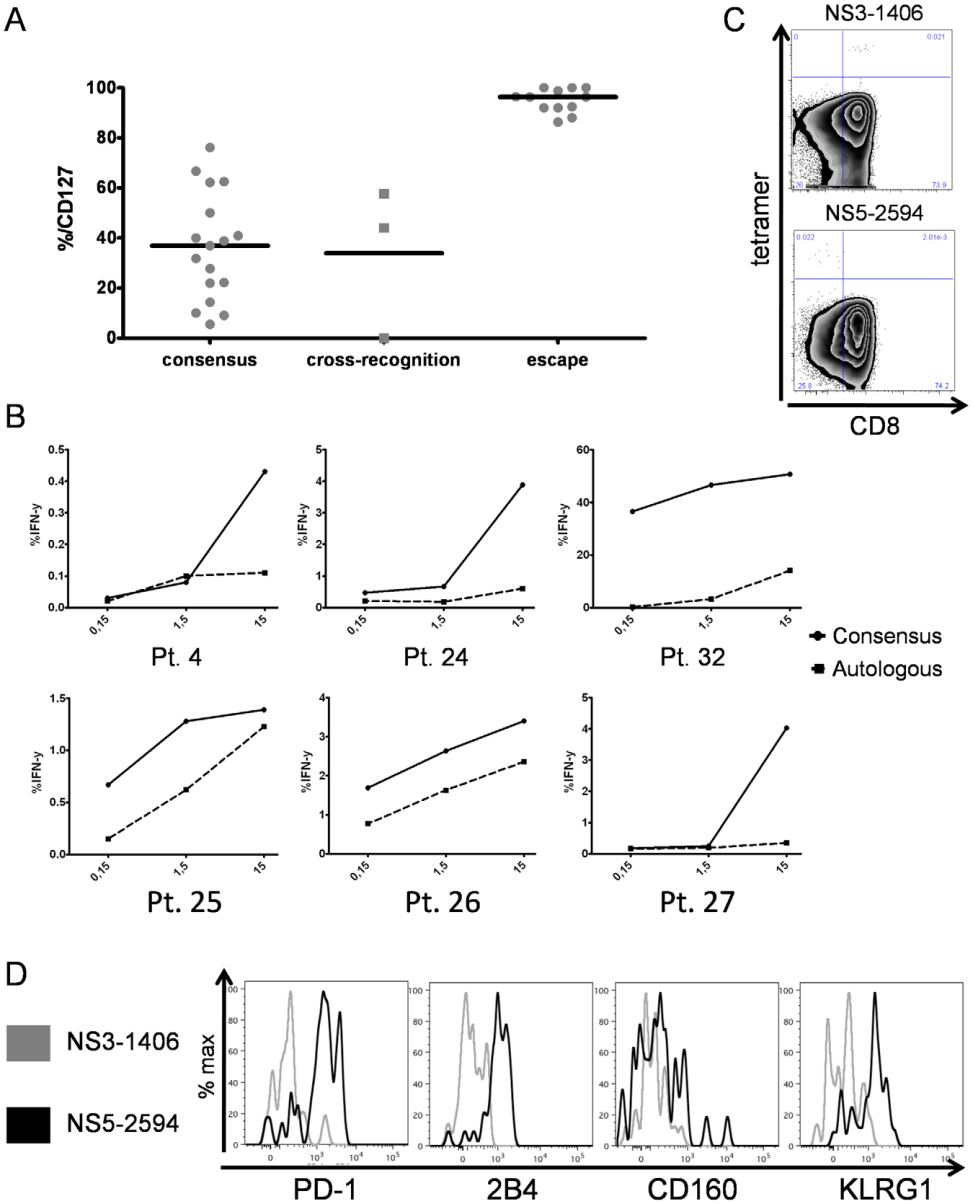
Inhibitory receptor expression is linked to recognition of viral antigen. (A) CD127 expression of HCV-specific CD8+ T cells grouped depending on the autologous virus sequence. Cross-recognition was defined as sequence variation from consensus that has been previously shown to be cross-recognized [39]. (B) PBMC of patients with variant autologous sequences (see table 2) were stimulated for IFN-γ expression after short term culture with different concentrations of consensus peptide (black line) or variant autologous peptide (dashed line). Representative diagrams are shown for pts. 4, 24–27 and 32. Frequency of IFN-γ expression is shown on the y axis, peptide concentration (µM) on the x axis. Viral escape is indicated by the strongly reduced IFN-γ production after stimulation with the autologous peptide sequence. (C) Divergent CD127 expression by HCV-specific CD8+ T cells on two epitopes from a single individual (pt. 4) targeting the NS3-1406 and NS5-2594 epitopes. (D) Overlay histograms of PD-1, 2B4, CD160 and KLRG1 expression on NS3-1406-specific CD8+ T cells (CD127hi) and NS5-2594-specific CD8+ T cells (CD127lo) from patient 4.

**Figure 9:**
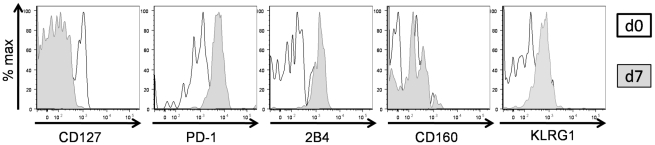
Upregulation of inhibitory receptor expression on CD127hi cells upon antigen stimulation. Comparison of CD127, PD-1, 2B4, CD160 and KLRG1 expression on HCV-specific CD8+ T cells prior and 7 days post stimulation are shown by overlay histograms for representative patient 24 (total n = 14).

In a final set of experiments, we set out to determine whether the differential expression of inhibitory receptors by phenotypically and functionally distinct HCV-specific CD8+ T cell populations is related to the autologous viral sequence present in a given patient. To address this issue, we sequenced the autologous viral sequences of genotype 1 patients corresponding to recognized CD8+ T cell epitopes (Table 2). Importantly, sequence variations from the genotype consensus sequence were significantly more prevalent within epitopes targeted by CD127hi HCV specific CD8+ T cells with a low expression of inhibitory receptors (p<0,0001)(Fig. 8A). We confirmed that these sequence variations represented viral escape in several patients by stimulating HCV-specific CD8+ T cells with peptides matched to both the autologous and consensus sequence. Indeed, as shown for six representative patients in Fig. 8B, HCV-specific CD8+ T cells produced high levels of IFN-γ upon stimulation with the consensus sequence peptide, but not when stimulated with the variant autologous peptide. Furthermore, proliferation of these HCV-specific CD8+ T cells was significantly reduced upon stimulation with the variant autologous peptide, indicating viral escape (data not shown). These results indicate that viral escape mutations are linked to a CD127hi phenotype whereas a CD127mid or CD127lo phenotype is linked to ongoing antigen recognition of the autologous virus sequence. To further test this hypothesis, we analyzed HCV-specific CD8+ T cells from patients with multiple immunodominant T cell responses that harbored consensus and variant sequences in different epitopes. Indeed, different expression profiles of inhibitory receptors and CD127 could be observed within the same patient depending on the virus sequence (patient 4, Fig. 8C, Table 2). Indeed, although both immunodominant responses were detectable at a comparable frequency (Fig. S3), all NS3-1406-specific CD8+ T cells expressed CD127, but only low levels of PD-1 (14.3%), 2B4 (4.17%), CD160 (2.09%) and KLRG1 (25.0%), whereas an opposite phenotype was observed on NS5-2594 specific CD8+ T cells, of which only 9,09% expressed CD127, but high levels of PD-1 (86.7%), 2B4 (81.8%), CD160 (27.3%) and KLRG1 (86.7%) (Fig. 8C, 8D). These results underline that the mechanisms responsible for inhibitory receptor coexpression operate in an epitope-specific manner depending on the autologous virus sequence. To further test the hypothesis that the low expression of inhibitory receptors and high expression of CD127 are indeed caused by absence of antigen triggering *in vivo*, we stimulated CD127hi HCV-specific CD8+ T cells with the consensus peptide *in vitro*. Importantly, this peptide specific stimulation induced a downregulation of CD127 and an upregulation of inhibitory receptor expression after a seven day culture (shown in Fig. 9). In sum, the downregulation of CD127 expression and upregulation of inhibitory receptors upon antigen stimulation suggest that the absence of antigen triggering *in vivo* is responsible for a CD127hi phenotype with a low expression of inhibitory receptors. In agreement with this hypothesis, we found a low expression of CD127 and a high expression of inhibitory receptors by virus-specific CD8+ T cells that targeted epitopes without sequence variations, indicating ongoing antigen recognition (Fig. 8A). These results also underscore the importance of evaluating autologous viral sequences in studies aimed at investigating the mechanisms of virus-specific CD8+ T cell failure.

**Table 2:** Epitope sequence analysis and correlation with CD127 expression.

Pt	gt	NS3-1073	%	CD127	NS3-1406	%	CD127	NS5-2594	%	CD127	NS5-2841	%	CD127
1	1b	CVNGVCWTV	0.06	lo									
2	1b	CVNGVCWTV	0.01	lo									
3	1b	n.a.	0.07	lo									
4	1a				KLVALG**V**NAV	0.02	hi	ALYDVVSKL	0.03	lo			
6	1a	CINGVCW**SI**	0.02	lo									
7	1a	CINGVCWT**A**	0.01	hi									
9	1b				KLSGLGLNAV	0.17	hi						
13	1b	n.a.	0.03	mid									
14	1b	CVNGVCWTV	0.02	lo									
15	1a	CINGVCWTV	0.12	lo									
16	1a	CINGVCWTV	0.05	lo				ALYDVVSKL	0.03	lo			
17	1a	n.a.	0.02	lo	n.a.	0.02	lo						
18	1b	CINGVCWTV	0.01	lo									
20	1a	CINGVCWTV	0.01	mid									
21	1b	C**I**NGVCWTV	0.02	mid									
22	1b	C**L**NGVCWTV	0.10	mid									
23	1b	CVNGVCWTV	0.02	lo									
24	1b										**V**RMIL**A**THF	0.18	hi
25	1a	CINGVCW**S**V	0.03	hi									
26	1a	CINGVCWTV	0.03	lo	KLVALG**V**NAV	0.04	mid						
27	1b										**V**RMI**M**MTHF	0.16	hi
28	1a	CINGVCWTV	0.04	lo									
29	1a	CINGVCWTV	0.27	lo									
30	1b	CVNGVCWTV	0.10	lo									
31	1b	C**VS**GVCWTV	0.13	hi									
32	1b	C**I**NGVCWTV	0.01	mid	KL**V**GLGLNAV	0.02	hi	ALYDVVS**K**L	0.20	hi			
33	1a	CINGVCWT**A**	0.05	hi	KLVALGINAV	0.07	mid	ALYDVVSKL	0.06	lo			
34	1a	CINGVCWTV	0.02	mid									
35	1a	CINGVCWTV	0.12	lo									
36	1a	CINGVCWTV	0.01	mid									
37	1b	CVNGVCWTV	0.03	lo									
38	1a	CINGVCWTV	0.03	lo									
Con	1a	CINGVCWTV			KLVALGINAV			ALYDVVSKL			ARMILMTHF		
Con	1b	CVNGVCWTV			KLSGLGLNAV			ALYDVVSTL			ARMILMTHF		

Autologous virus sequences were obtained from patients with genotype 1 with detectable HCV-specific CD8+ T cell responses targeting immunodominant epitopes. Level of CD127 on T cells recognizing these epitopes is indicated next to the tetramer frequency and the respective sequence (hi: >80% CD127 expression, mid: 50–80% CD127 expression, lo: <50% CD127 expression). The consensus sequences for genotype 1a and 1b are shown below. Differing amino acids from variant autologous sequences are highlighted in bold. Abb.: Con, consensus sequence; gt, HCV genotype; n.a., not available; Pt, patient.

## Discussion

Here, we have investigated the expression of inhibitory receptors by virus-specific CD8+ T cells during chronic HCV infection and their association with CD127 expression, proliferative capacity, T cell differentiation and antigen recognition. We found a specific pattern of expression of inhibitory receptors by HCV-specific CD8+ T cells. Indeed, a large fraction of the HCV-specific CD8+ T cell responses detectable in our cohort coexpressed 2B4, CD160 and KLRG1 next to PD1. These results are interesting in the light of a recent study by Blackburn et al. demonstrating a clear association between a specific expression pattern of inhibitory receptors including 2B4, CD160, LAG-3 and CTLA-4 next to PD-1 and severe exhaustion of virus-specific CD8+ T cells in LCMV infection in mice [9]. Interestingly, we did not observe a significant expression of LAG-3 and CTLA-4 on HCV-specific CD8+ T cells in our study. However, these results are not surprising since it has previously been shown that CTLA-4 expression by HCV-specific CD8+ T cells is only detectable to some degree in the liver, but not in the blood of chronically infected patients [5]. In addition, a gene expression analysis of exhausted CD8+ T cells in mice revealed that LAG-3 and CTLA-4 coexpression showed a weaker association with PD-1 compared to 2B4 and CD160 coexpression [12]. Thus, these combined results indicate a dynamic hierarchical pattern of inhibitory receptor expression by exhausted CD8+ T cells with prominent roles of 2B4 and CD160 [12]. Accordingly, our results indicate that a significant fraction of HCV-specific CD8+ T cells display typical markers of exhaustion. Importantly, HCV-specific CD8+ T cell exhaustion defined as a loss of CD8+ T cell function, e.g. proliferation and cytokine secretion has been reported by several groups [4], [5], [20], [21], [22]. Our findings implicate that therapeutic targeting of exhausted CD8+ T cells during chronic HCV infection may require not only the blockade of one inhibitory receptor pathway, but rather a cocktail of antibodies targeting multiple inhibitory pathways [6]. In line with these findings, we observed only a 2.2fold increase in proliferation of exhausted CD127lo HCV-specific CD8+ T cells upon PD-1/PD-L1 blockade (Fig. 4D). An even smaller increase in proliferation due to PD-1/PD-L1 blockade was seen in CD127hi HCV-specific CD8+ T cells, however, this may be explained by the finding that these cells proliferate vigorously in the absence of blockade, indicating that they are not significantly inhibited (Fig. 4C–E and [17]). Clearly, additional studies should address the effect of 2B4, CD160 and KLRG1 receptor blockade on exhausted T cells as soon as suitable blocking reagents are available.

2B4 is a coregulatory receptor capable of mediating activatory and inhibitory signals in CD8+ T cells [23]. It was previously considered as a predominantly activating receptor in humans [24]. However, a recent study addressed the dual roles of murine and human 2B4 receptor signaling and found that both human and murine 2B4 can exert both inhibitory or activatory receptor functions [10]. Importantly, high levels of 2B4 surface expression were linked to inhibitory 2B4 function, whereas low levels of 2B4 surface expression promoted activatory 2B4 signals [10]. Based on the data analyzed in our study, we cannot exclude that ligation of 2B4 on HCV-specific CD8+ T cells may result in a costimulatory signal. However, we used a strict gating strategy to determine 2B4 positivity only on cells with a high surface expression level of 2B4 (Fig. 2B and data not shown), indicating that 2B4 may act as an inhibitory receptor on these cells. Further studies will be needed to clarify whether 2B4 expression on virus-specific CD8+ T cells, indeed, promotes inhibitory signals.

Of note, we observed significant levels of KLRG1 expression on HCV-specific CD8+ T cells (Fig. 1,2). KLRG1 is up-regulated by virus-specific CD8+ T cells upon repetitive antigenic stimulation and frequently used as a marker for T cell differentiation [16], [25]. Although KLRG1 expression was not shown to be strongly associated with PD-1 coexpression by exhausted CD8+ T cells in mice it was, however, identified on a significant fraction of exhausted CD8+ T cells [9], [12]. Since the up-regulation of KLRG1 is induced by repetitive antigen stimulation, it is well possible that KLRG1 expression might not directly reflect T cell exhaustion but rather ongoing antigen recognition, that, however, by itself is thought to be the prerequisite for the development of CD8+ T cell exhaustion [26], [27].

Our results also show that the expression level of the inhibitory receptors PD-1, 2B4, CD160 and KLRG1 is linked to a specific stage of cell differentiation. Recently, a linear model of human CD8+ T cell differentiation has been developed that distinguishes between naïve, early, intermediate and late differentiated cells [28]. The differentiation status of CD8+ T cells is influenced by the history of antigenic stimulation, clonal cell division, telomere length and proliferative capacity of CD8+ T cells [18]. Here, we found that the expression of PD-1 was highest on CD8+ T cells with an intermediate T cell differentiation phenotype. In contrast, 2B4, CD160 and KLRG1 were differentially expressed by late differentiated CD8+ T cells (Fig. 5). However, the highest level of coexpression of all inhibitory receptors was observed within the intermediate T cell differentiation stage (Fig. 6), indicating that exhaustion of CD8+ T cells is most likely linked to this specific subset. It remains unclear whether the coexpression of inhibitory receptors 2B4, CD160 and KLRG1 by late differentiated cells in the absence of PD-1 may also define a specific subset of exhausted CD8+ T cells. However, this is rather unlikely since coexpression of inhibitory receptors 2B4 and CD160 by late T cell differentiated T cells has been shown to define a functional cytotoxic CD8+ T cell population [24], [29].

Another important finding of our study is that coexpression of inhibitory receptors on HCV-specific CD8+ T cells is linked to the expression level of CD127. We have previously described the existence of subsets of HCV-specific CD8+ T cells with distinct functional properties that can be distinguished by the level of CD127 expression [17]. Here, we classified HCV-specific CD8+ T cells depending on the level of CD127 expression into three groups: CD127lo, CD127mid and CD127hi cells. CD127lo HCV-specific CD8+ T cells express markers associated with effector cells and are dysfunctional in terms of proliferation whereas CD127hi cells phenotypically and functionally resemble memory CD8+ T cells [17]. These memory-like properties are also reflected by an increased expression of antiapoptotic molecules [30], [31](Fig. S3). Here, we found that high coexpression of inhibitory receptors next to PD-1 was restricted to CD127lo HCV-specific CD8+ T cells with impaired proliferative capacity that could be reinvigorated upon PD-1/PD-L1 signaling blockade, thus, correlating well with the impaired functional state of these cells. We also found that HCV-specific CD8+ T cells do not represent a homogenously differentiated CD8+ T cell population. This novel finding was made possible through the use of polychromatic flow cytometry that allowed us to simultaneously stain HCV-specific CD8+ T cells for the set of differentiation markers recommended by Appay et al. [18] and extends previous reports regarding the differentiation of HCV-specific CD8+ T cells that used conventional 4-color flow cytometry [18], [28]. Of note, the differentiation stage of HCV-specific CD8+ T cells was significantly different for CD127hi, CD127mid and CD127lo cells (Fig. 7). In addition, the intermediate differentiation of HCV-specific CD8+ T cells characterized by the coexpression of multiple inhibitory receptors (as observed prominently for CD127lo cells) correlates with the coexpression of inhibitory receptors in intermediate differentiation subsets observed on the general CD8+ T cell population. Since these results underline the existence of phenotypically and functionally very different HCV-specific CD8+ T cell populations during chronic HCV infection that can be defined by the differential expression of CD127 and inhibitory receptors, respectively, it will be important to distinguish between these populations in future studies analyzing HCV-specific CD8+ T cells.

Another important finding of our study is that the expression of inhibitory receptors by virus-specific CD8+ T cells is associated with ongoing antigen recognition and the absence of viral escape. Since we could not analyze the virus sequence that initially infected our patients, and we thus could not observe the development of escape mutations as has been elegantly shown in recent longitudinal studies in acute HCV infection [32], [33], we assumed that the majority of patients was infected with the genotype consensus sequence (table 2). This approach has some limitations, for example it cannot be ruled out that remnants of a prior infection with an alternate genotype or varying epitope sequence confounded our analysis (for example, the sequence of the autologous epitope matches the genotype consensus sequence in one CD127hi patient (the NS3-1406 epitope in subject 9)). In addition, it is impossible to dissect whether a variant epitope that matches the heterologous genotype sequence that is targeted by a CD127hi T cell response represents viral escape or whether the T cell response represents the remnant of a prior resolved infection with the alternate genotype (e.g., the NS5-2594 epitope in subject 32). Clearly, these issues can only be addressed in cohorts of HCV-infected patients, in which the sequence of the inoculum and information about the T cell response prior to infection is known. However, the clear association of high levels of CD127 expression on virus-specific CD8+ T cells with the presence of autologous sequence variations from the consensus epitopes, the reduced IFN-γ production and reduced proliferation of CD8+ T cells upon stimulation with variant but not with consensus peptides supports our approach in the majority of cases. Of note, we also found a clear association between the expression level of inhibitory receptors next to PD1 and the absence of sequence variations indicating ongoing antigen recognition. These results are in agreement with recent studies showing that high levels of specific antigen drive CD8+ T cell exhaustion [27] and that exhaustion of CD8+ T cells can be prevented by the emergence of viral escape mutations that abrogate epitope recognition [34]. In line with these findings, emergence of viral sequence variations was associated with a reduction of PD-1 expression and upregulation of CD127 expression in acute and chronic HCV infection [32], [33] and HIV infection [35]. It is also interesting to note that we observed sequence variations in about half of the targeted T cell epitopes. Although the number of HCV-specific CD8+ T cell epitopes analyzed in our study was limited, and it is thus hard to make firm conclusions about the general frequencies of viral escape among HCV-specific CD8+ T cell epitopes, our results are in agreement with recent studies that also have shown the presence of viral escape mutations in about 50% of targeted CD8+ T cell epitopes [36], [37]. Thus, both mechanisms of CD8+ T cell failure, viral escape and T cell exhaustion, defined by the coexpression of multiple inhibitory receptors, seem to contribute significantly to the ineffective viral control and they can be easily identified by specific surface expression patterns.

In sum, our results show that the coexpression of inhibitory receptors by exhausted HCV-specific CD8+ T cells is linked to CD127 expression, proliferative capacity, the stage of T cell differentiation and ongoing antigen recognition. Thus, a complex interplay of immunological and virological factors determines T cell exhaustion in human chronic viral infection. These findings have also important implications for the rational design of immunotherapeutic treatment strategies in chronic HCV infection since only exhausted CD8+ T cells still recognize the present viral antigens and should thus be targeted by immunomodulatory strategies.

## Materials and Methods

### Study group

38 patients with chronic HCV infection presenting at the outpatient hepatology clinic of the University Hospital Freiburg with detectable HCV-specific CD8+ tetramer responses were included in the study after obtaining written informed consent from the patients and approval by the ethics committee of the Albert-Ludwigs-Universität, Freiburg. All investigations have been conducted according to the principles expressed in the Declaration of Helsinki. The characteristics of the study population are included in table 1.

### HCV viral load and sequencing

Detection of HCV viral load and epitope sequencing was performed as previously described [37].

### Lymphocyte isolation

Lymphocytes were analyzed from patient blood as previously described [17].

### Peptides and tetramers

Peptides corresponding to immunodominant HLA-A2- and HLA-B27- epitopes (sequences CINGVCWTV, KLVALGINAV, ALYDVVTKL, ARMILMTHF) and variant autologous patient sequences were obtained from Biosynthan, Berlin, Germany. These peptides were dissolved in 100% dimethyl sulfoxide (Sigma-Aldrich, Germany) at 20 mg/ml and further diluted to 1 mg/ml with RPMI 1640 (Gibco) before use. HLA-A2- and HLA-B27-tetramers were obtained from the National Tetramer Core Facility at Emory University, Atlanta. Influenza-specific pentamers containing the immunodominant GILGFVFTL peptide were purchased from ProImmune, Oxford, UK.

### Antibodies

The following reagents were used for polychromatic stainings: anti-2B4-FITC, anti-CD27-APC-eFluor780, anti-CD45RA-PerCP-Cy5.5, anti-PD-1-PE, anti-CD127-APC Alexa Fluor 750, anti-CD127-APC-eFluor 780, anti-CD127-Pacific Blue, anti-IgM-PerCPeFluor710, streptavidin-eFluorV450 (Ebioscience), anti-Bcl-2-PE, anti-CCR7-PE-Cy7, anti-CD28-FITC, anti-CD38-PE, anti-CD8-APC H7, anti-CD8 AmCyan (BD Biosciences), anti-CD127-PE, anti-CD160-PE, anti-CD57-FITC (Beckman Coulter), anti-CTLA-4-FITC (R&D), anti-CD3-PerCP, anti-CD160-FITC, purified anti-CD160 (BioLegend), anti-LAG-3-Atto488 (Alexxis), anti-LAG-3 FITC (LifeSpan BioSciences), anti-LAG-3-PE (R&D). Viaprobe (7-AAD, BD Biosciences) was used for dead cell exclusion. The anti-KLRG1-AlexaFluor488 and anti-KLRG1-biotin antibodies were generated as previously described [16].

### Polychromatic flow cytometry

Tetramer and antibody staining was performed as previously described [17]. For the multi-inhibitory receptor staining, the cells were first stained with the CD160-IgM-pure antibody, then with the anti-IgM-PerCP secondary antibody, then stained with the KLRG1-biotin antibody followed with the streptavidin-eFluorV450 antibody. Cells were then incubated with the tetramer followed by the staining with the fluorophore-conjugated antibodies (CD8-AmCyan, 2B4-FITC, PD-1-PE, CD127-APC-eFluor780). Incubation time was 15 min for each staining step and cells were washed twice in between the addition of staining reagents.

Samples were acquired on a FACS Canto II flow cytometer (BD Biosciences) and analyzed with FlowJo v8.8.6 software (TreeStar Inc.). Gates for positivity in polychromatic panels were determined by fluorescence-minus-one control stains, as recommended [38].

### Proliferation and blockade assays

2*10∧6 freshly isolated PBMCs were labelled with 40 µM Pacific Blue succinimidyl ester (PBSE, Invitrogen), and stimulated with 10 µM peptide in the presence of 20 IU/ml rhIl-2 (Roche) in 1 ml complete medium (RPMI 1640 containing 10% fetal calf serum, 1% streptomycin/penicillin, and 1.5% Hepes buffer 1 mol/L). Cells were incubated at 37°C in the presence or absence of 10 µg/ml anti-PD-L1 (Ebioscience) for one week, subsequently stained and analyzed by polychromatic flow cytometry. PD-L1 blockade-dependent gain of proliferation was determined by the ratio: frequency of HCV-specific CD8+ T cells after peptide stimulation and PD-L1 blockade divided by the frequency after peptide stimulation alone. The proliferation index was calculated by dividing the frequency of HCV-specific CD8+ T cells after stimulation by the frequency *ex vivo*.

### Statistical analysis

Statistical analysis was performed using GraphPad Prism 5 software (GraphPad Prism Software, Inc.). The statistical tests used were ANOVA 1way analysis of variance followed by Newman-Keuls Multiple Comparison Test (Fig. 3,4,7), and Mann-Whitney U test (Fig. 8).

## Supporting Information

Figure S1 Gating strategy for the assessment of CD127 frequencies. Virus-specific CD8+ T cells were determined by tetramer staining and thresholds for CD127 positivity defined by FMO controls. Representative pseudocolor plots are shown for (A) CD127hi HCV-specific CD8+ T cells (pt. 4-NS3-1406) and (B) CD127lo HCV-specific CD8+ T cells (pt. 4-NS5-2594).
(0.43 MB TIF)

Figure S2 Multi-inhibitory receptor analysis of HCV-specific CD8+ T cells. The co-expression of PD-1, 2B4, CD160 and KLRG1 on CD8+tetramer+ T cells was determined as follows: A) The tetramer+ HCV-specific CD8+ T cell population was gated on CD8+ T cells in the lymphocyte gate. Gates for CD127 and inhibitory receptor expression were determined on the total CD8+ population in comparison to FMO controls. B) These gates were applied to the tetramer+ population and a Boolean matrix used to determine the number of inhibitory receptors expressed by individual HCV-specific CD8+ T cells.
(0.80 MB TIF)

Figure S3 Analysis of Bcl-2 expression. In a total of 8 patients, Bcl-2 expression on HCV-specific CD8+ T cells was analyzedAnalysis of Bcl-2 expression. In a total of 8 patients, Bcl-2 expression on HCV-specific CD8+ T cells was analyzed after cell permeabiliztion. Original data plots from patient 4 are shown and the MFI of tetramer-positive cells is displayed. Bcl-2 expression correlated with the level of CD127 expression. The coexpression of inhibitory receptors of the displayed HCV-specific CD8+ T cells is shown in Fig. 3C. after cell permeabiliztion. Original data plots from patient 4 are shown and the MFI of tetramer-positive cells is displayed. Bcl-2 expression correlated with the level of CD127 expression. The coexpression of inhibitory receptors of the displayed HCV-specific CD8+ T cells is shown in Fig. 3C.
(0.29 MB TIF)

## References

[1] Rehermann B (2009). Hepatitis C virus versus innate and adaptive immune responses: a tale of coevolution and coexistence.. J Clin Invest.

[2] Barber DL, Wherry EJ, Masopust D, Zhu B, Allison JP (2006). Restoring function in exhausted CD8 T cells during chronic viral infection.. Nature.

[3] Radziewicz H, Ibegbu CC, Fernandez ML, Workowski KA, Obideen K (2007). Liver-infiltrating lymphocytes in chronic human hepatitis C virus infection display an exhausted phenotype with high levels of PD-1 and low levels of CD127 expression.. J Virol.

[4] Penna A, Pilli M, Zerbini A, Orlandini A, Mezzadri S (2007). Dysfunction and functional restoration of HCV-specific CD8 responses in chronic hepatitis C virus infection.. Hepatology.

[5] Nakamoto N, Kaplan DE, Coleclough J, Li Y, Valiga ME (2008). Functional restoration of HCV-specific CD8 T cells by PD-1 blockade is defined by PD-1 expression and compartmentalization.. Gastroenterology.

[6] Nakamoto N, Cho H, Shaked A, Olthoff K, Valiga ME (2009). Synergistic reversal of intrahepatic HCV-specific CD8 T cell exhaustion by combined PD-1/CTLA-4 blockade.. PLoS Pathog.

[7] Kasprowicz V, Schulze Zur Wiesch J, Kuntzen T, Nolan BE, Longworth S (2008). High level of PD-1 expression on hepatitis C virus (HCV)-specific CD8+ and CD4+ T cells during acute HCV infection, irrespective of clinical outcome.. J Virol.

[8] Bowen DG, Shoukry NH, Grakoui A, Fuller MJ, Cawthon AG (2008). Variable patterns of programmed death-1 expression on fully functional memory T cells after spontaneous resolution of hepatitis C virus infection.. J Virol.

[9] Blackburn SD, Shin H, Haining WN, Zou T, Workman CJ (2009). Coregulation of CD8+ T cell exhaustion by multiple inhibitory receptors during chronic viral infection.. Nat Immunol.

[10] Chlewicki LK, Velikovsky CA, Balakrishnan V, Mariuzza RA, Kumar V (2008). Molecular basis of the dual functions of 2B4 (CD244).. J Immunol.

[11] Cai G, Anumanthan A, Brown JA, Greenfield EA, Zhu B (2008). CD160 inhibits activation of human CD4+ T cells through interaction with herpesvirus entry mediator.. Nat Immunol.

[12] Wherry EJ, Ha SJ, Kaech SM, Haining WN, Sarkar S (2007). Molecular signature of CD8+ T cell exhaustion during chronic viral infection.. Immunity.

[13] Henson SM, Franzese O, Macaulay R, Libri V, Azevedo RI (2009). KLRG1 signaling induces defective Akt (ser473) phosphorylation and proliferative dysfunction of highly differentiated CD8+ T cells.. Blood.

[14] Rosshart S, Hofmann M, Schweier O, Pfaff AK, Yoshimoto K (2008). Interaction of KLRG1 with E-cadherin: new functional and structural insights.. Eur J Immunol.

[15] Voehringer D, Blaser C, Brawand P, Raulet DH, Hanke T (2001). Viral infections induce abundant numbers of senescent CD8 T cells.. J Immunol.

[16] Thimme R, Appay V, Koschella M, Panther E, Roth E (2005). Increased expression of the NK cell receptor KLRG1 by virus-specific CD8 T cells during persistent antigen stimulation.. J Virol.

[17] Bengsch B, Spangenberg HC, Kersting N, Neumann-Haefelin C, Panther E (2007). Analysis of CD127 and KLRG1 expression on hepatitis C virus-specific CD8+ T cells reveals the existence of different memory T-cell subsets in the peripheral blood and liver.. J Virol.

[18] Appay V, van Lier RA, Sallusto F, Roederer M (2008). Phenotype and function of human T lymphocyte subsets: consensus and issues.. Cytometry A.

[19] Sauce D, Almeida JR, Larsen M, Haro L, Autran B (2007). PD-1 expression on human CD8 T cells depends on both state of differentiation and activation status.. AIDS.

[20] Spangenberg HC, Viazov S, Kersting N, Neumann-Haefelin C, McKinney D (2005). Intrahepatic CD8+ T-cell failure during chronic hepatitis C virus infection.. Hepatology.

[21] Wedemeyer H, He XS, Nascimbeni M, Davis AR, Greenberg HB (2002). Impaired effector function of hepatitis C virus-specific CD8+ T cells in chronic hepatitis C virus infection.. J Immunol.

[22] Golden-Mason L, Palmer B, Klarquist J, Mengshol JA, Castelblanco N (2007). Upregulation of PD-1 expression on circulating and intrahepatic hepatitis C virus-specific CD8+ T cells associated with reversible immune dysfunction.. J Virol.

[23] Ma CS, Nichols KE, Tangye SG (2007). Regulation of cellular and humoral immune responses by the SLAM and SAP families of molecules.. Annu Rev Immunol.

[24] Speiser DE, Colonna M, Ayyoub M, Cella M, Pittet MJ (2001). The activatory receptor 2B4 is expressed in vivo by human CD8+ effector alpha beta T cells.. J Immunol.

[25] Joshi NS, Cui W, Chandele A, Lee HK, Urso DR (2007). Inflammation directs memory precursor and short-lived effector CD8(+) T cell fates via the graded expression of T-bet transcription factor.. Immunity.

[26] Virgin HW, Wherry EJ, Ahmed R (2009). Redefining chronic viral infection.. Cell.

[27] Mueller SN, Ahmed R (2009). High antigen levels are the cause of T cell exhaustion during chronic viral infection.. Proc Natl Acad Sci U S A.

[28] Appay V, Dunbar PR, Callan M, Klenerman P, Gillespie GM (2002). Memory CD8+ T cells vary in differentiation phenotype in different persistent virus infections.. Nat Med.

[29] Rey J, Giustiniani J, Mallet F, Schiavon V, Boumsell L (2006). The co-expression of 2B4 (CD244) and CD160 delineates a subpopulation of human CD8+ T cells with a potent CD160-mediated cytolytic effector function.. Eur J Immunol.

[30] Badr G, Bedard N, Abdel-Hakeem MS, Trautmann L, Willems B (2008). Early interferon therapy for hepatitis C virus infection rescues polyfunctional, long-lived CD8+ memory T cells.. J Virol.

[31] Golden-Mason L, Burton JR, Castelblanco N, Klarquist J, Benlloch S (2006). Loss of IL-7 receptor alpha-chain (CD127) expression in acute HCV infection associated with viral persistence.. Hepatology.

[32] Kasprowicz V, Kang YH, Lucas M, Schulze Zur Wiesch J, Kuntzen T (2009). HCV sequence variation induces an HCV-specific T cell phenotype analogous to spontaneous resolution.. J Virol.

[33] Rutebemberwa A, Ray SC, Astemborski J, Levine J, Liu L (2008). High-programmed death-1 levels on hepatitis C virus-specific T cells during acute infection are associated with viral persistence and require preservation of cognate antigen during chronic infection.. J Immunol.

[34] Blattman JN, Wherry EJ, Ha SJ, van der Most RG, Ahmed R (2009). Impact of epitope escape on PD-1 expression and CD8 T-cell exhaustion during chronic infection.. J Virol.

[35] Streeck H, Brumme ZL, Anastario M, Cohen KW, Jolin JS (2008). Antigen load and viral sequence diversification determine the functional profile of HIV-1-specific CD8+ T cells.. PLoS Med.

[36] Cox AL, Mosbruger T, Mao Q, Liu Z, Wang XH (2005). Cellular immune selection with hepatitis C virus persistence in humans.. J Exp Med.

[37] Neumann-Haefelin C, Timm J, Spangenberg HC, Wischniowski N, Nazarova N (2008). Virological and immunological determinants of intrahepatic virus-specific CD8+ T-cell failure in chronic hepatitis C virus infection.. Hepatology.

[38] Mahnke YD, Roederer M (2007). Optimizing a multicolor immunophenotyping assay.. Clin Lab Med.

[39] Fytili P, Dalekos GN, Schlaphoff V, Suneetha PV, Sarrazin C (2008). Cross-genotype-reactivity of the immunodominant HCV CD8 T-cell epitope NS3-1073.. Vaccine.

