# Supplementary figures and images for: Coexpression of PD-1, 2B4, CD160 and KLRG1 on Exhausted HCV-Specific CD8+ T Cells Is Linked to Antigen Recognition and T Cell Differentiation
Source: PLoS Pathog. 2010 Jun 10;6(6):e1000947. doi: 10.1371/journal.ppat.1000947 (PMC2883597; doi:10.1371/journal.ppat.1000947)

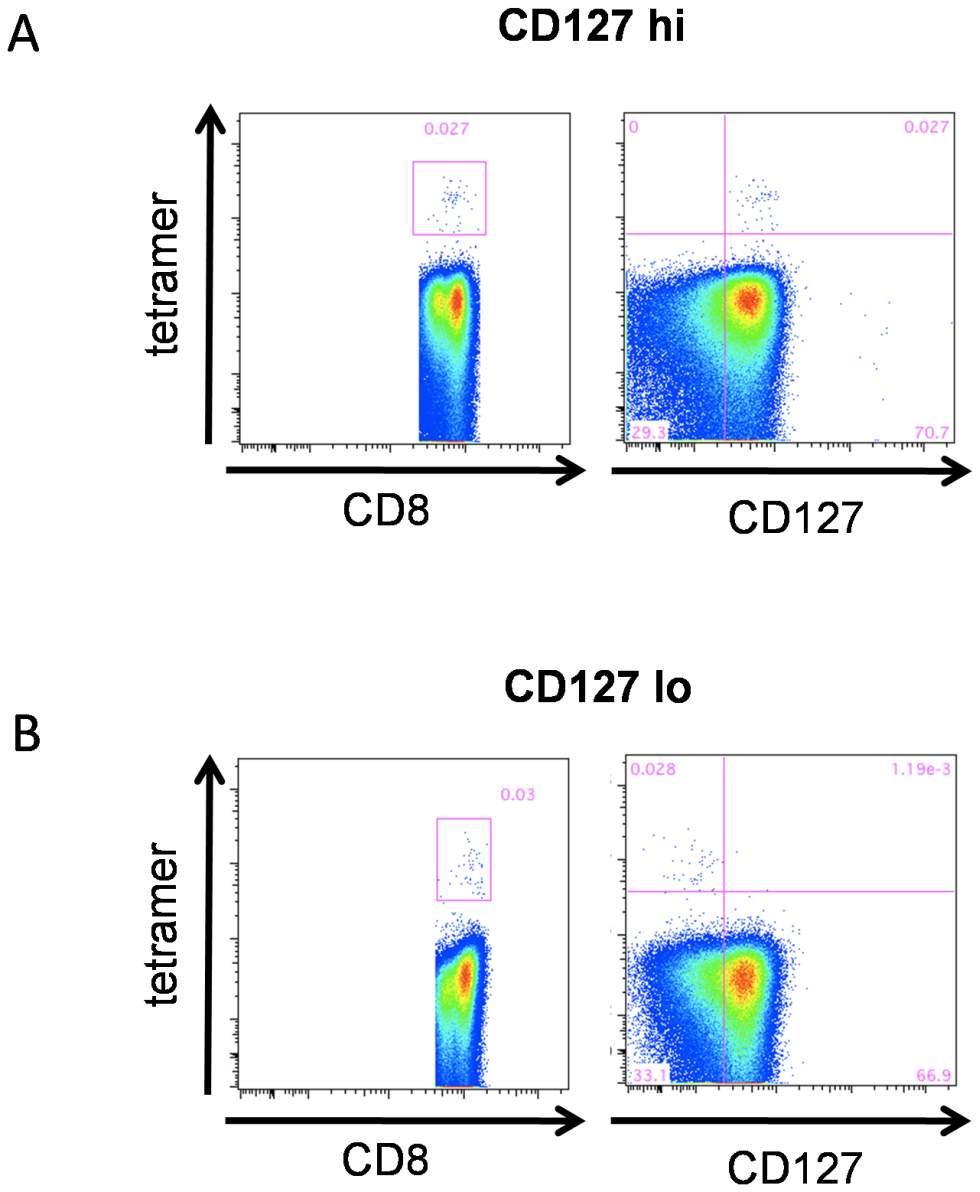

Supplement: Figure S1 — Gating strategy for the assessment of CD127 frequencies. Virus-specific CD8+ T cells were determined by tetramer staining and thresholds for CD127 positivity defined by FMO controls. Representative pseudocolor plots are shown for (A) CD127hi HCV-specific CD8+ T cells (pt. 4-NS3-1406) and (B) CD127lo HCV-specific CD8+ T cells (pt. 4-NS5-2594). (0.43 MB TIF) [file ppat.1000947.s001.tif]

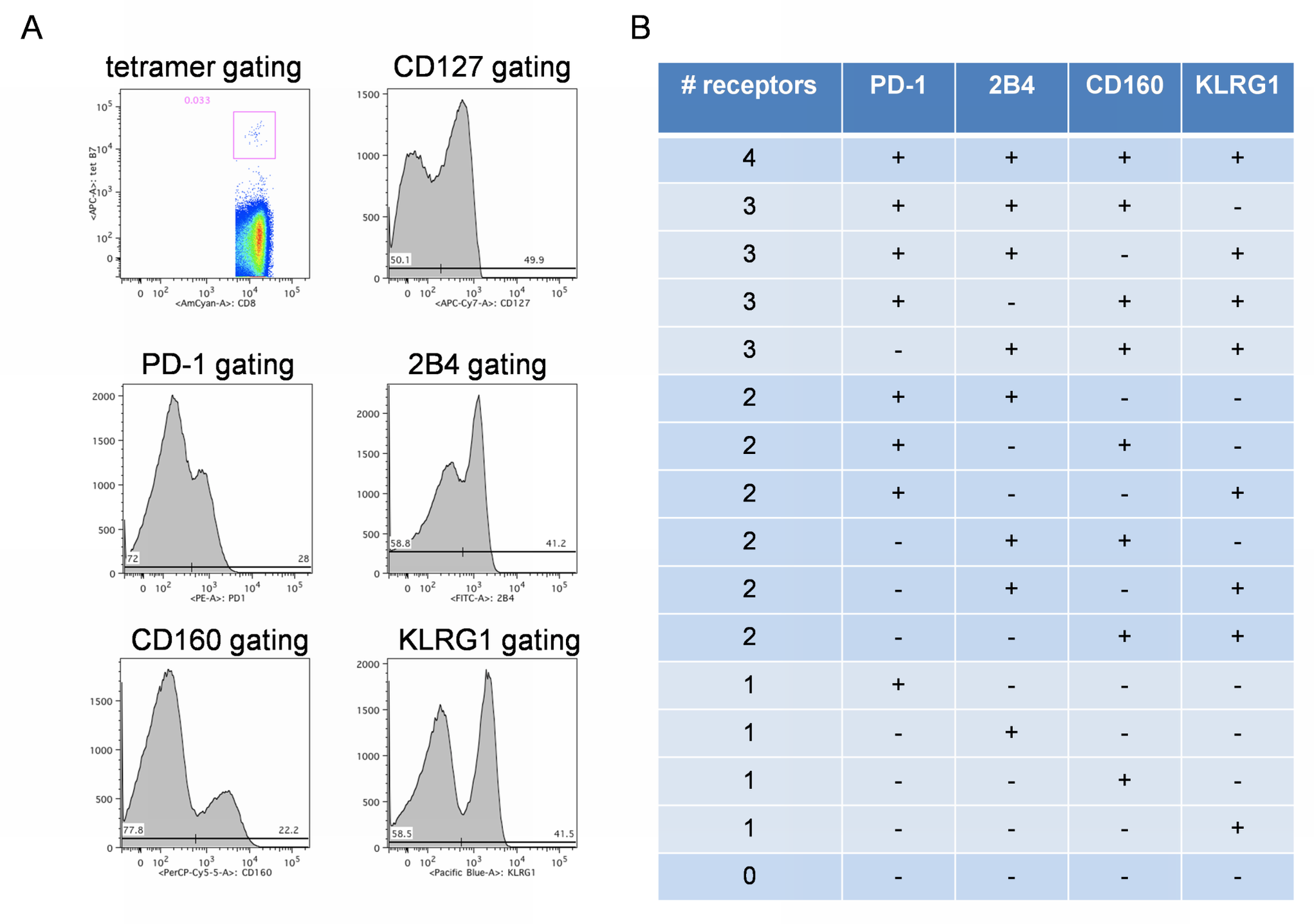

Supplement: Figure S2 — Multi-inhibitory receptor analysis of HCV-specific CD8+ T cells. The co-expression of PD-1, 2B4, CD160 and KLRG1 on CD8+tetramer+ T cells was determined as follows: A) The tetramer+ HCV-specific CD8+ T cell population was gated on CD8+ T cells in the lymphocyte gate. Gates for CD127 and inhibitory receptor expression were determined on the total CD8+ population in comparison to FMO controls. B) These gates were applied to the tetramer+ population and a Boolean matrix used to determine the number of inhibitory receptors expressed by individual HCV-specific CD8+ T cells. (0.80 MB TIF) [file ppat.1000947.s002.tif]

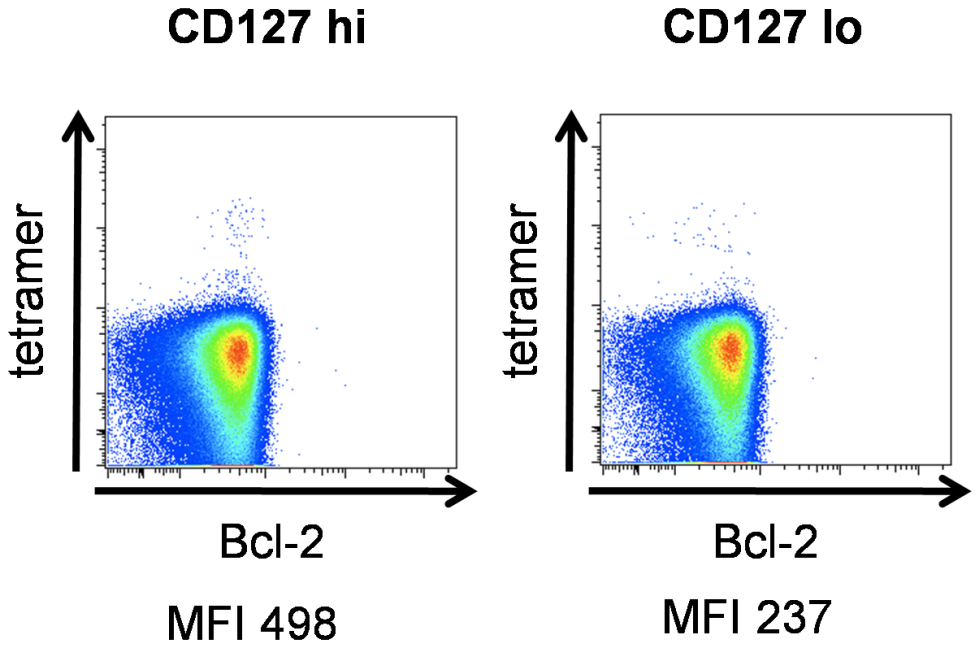

Supplement: Figure S3 — Analysis of Bcl-2 expression. In a total of 8 patients, Bcl-2 expression on HCV-specific CD8+ T cells was analyzedAnalysis of Bcl-2 expression. In a total of 8 patients, Bcl-2 expression on HCV-specific CD8+ T cells was analyzed after cell permeabiliztion. Original data plots from patient 4 are shown and the MFI of tetramer-positive cells is displayed. Bcl-2 expression correlated with the level of CD127 expression. The coexpression of inhibitory receptors of the displayed HCV-specific CD8+ T cells is shown in Fig. 3C. after cell permeabiliztion. Original data plots from patient 4 are shown and the MFI of tetramer-positive cells is displayed. Bcl-2 expression correlated with the level of CD127 expression. The coexpression of inhibitory receptors of the displayed HCV-specific CD8+ T cells is shown in Fig. 3C. (0.29 MB TIF) [file ppat.1000947.s003.tif]
